# Perioperative cancer cell dissemination detected with a real-time RT-PCR assay for EpCAM is not associated with worse prognosis in pancreatic ductal adenocarcinoma

**DOI:** 10.1186/1471-2407-11-47

**Published:** 2011-01-31

**Authors:** Gregory Sergeant, Tania Roskams, Jos van Pelt, François Houtmeyers, Raymond Aerts, Baki Topal

**Affiliations:** 1 Department of Abdominal Surgery, University Hospital Leuven, Herestraat 49, B-3000 Leuven, Belgium; 2 Department of Pathology, University Hospital Leuven, Herestraat 49, B-3000 Leuven, Belgium; 3 Department of Hepatology, University Hospital Leuven, Herestraat 49, B-3000 Leuven, Belgium; 4 CEMOL, University Hospital Leuven, Herestraat 49, B-3000 Leuven, Belgium

## Abstract

**Background:**

Epithelial cell adhesion molecule (EpCAM) has been used as surrogate marker for the quantification of circulating tumour cells (CTC). Our aim was to prospectively study the value of a real-time RT-PCR assay for EpCAM detection in the peripheral blood and peritoneal cavity of patients undergoing pancreatectomy for pancreatic ductal adenocarcinoma (PDAC).

**Methods:**

From 48 patients with PDAC (40 resectable, 8 unresectable) and 10 patients with chronic pancreatitis undergoing pancreatectomy 10 ml of venous blood was drawn preoperatively (PB) and postoperatively (POB, day 1 (D1B), day 7 (D7B) and after 6 weeks (6WB). Of all patients undergoing pancreatectomy, 40 ml peritoneal lavage fluid was taken preoperatively and postoperatively. A real-time RT-PCR assay (TaqMan, ABI Prism 7700) was developed for the detection of EpCAM mRNA. To discriminate between EpCAM-positive and negative samples a cut-off was applied. Median postoperative follow-up was 24.0 months (range: 0.7 - 41.3).

**Results:**

PB was EpCAM-positive (+) in 25% of patients versus 65% of patients in POB (p < 0.0001). EpCAM(+) was noted at D1B, D7B and 6WB was found in 28.6%, 23.1% and 23.5% of patients respectively. Preoperative peritoneal lavage fluid was EpCAM(+) in 10.3% versus 53.8% of patients postoperatively (p < 0.0001).

At none of the time-points, an association was found between EpCAM positivity in blood and/or peritoneal cavity and cancer-specific or disease-free survival. Also, no significant associations were found between clinicopathological variables and perioperative EpCAM positivity.

**Conclusions:**

Despite a significant increase in EpCAM counts in postoperative blood and peritoneal lavage fluid this was not associated with worse prognosis after pancreatectomy for PDAC.

**Trial registration:**

Clinicaltrials.gov NCT00495924

## Background

The vast majority of patients suffering from solid organ tumours, such as pancreatic cancer, ultimately die from metastases that develop at sites far from the primary tumour. These distant organ metastases are the end-results of a mainly haematogenous cancer cell dissemination from the primary tumour. Pancreatic cancer or pancreatic ductal adenocarcinoma (PDAC) is one of the most lethal malignancies in humans. In selected patients with localised PDAC, surgical resection is the only treatment offering long-time survival. Despite the curative intent of surgical resection, cancer recurrence in the liver and/or the peritoneal cavity develops within two years after pancreatic surgery in over 60% of patients [1]. Therefore, recent evidence supports that surgical resection of solid tumours could promote tumour escape [2,3]. Indeed, intraoperative tumour manipulation results in detachment of tumour cells that may cause metastases [4]. Additionally, the postoperative phase is characterized by transient changes in the immune system hampering the anti-tumoural response and rendering the host more susceptible to metastasis. The release of certain mediators (e.g. IL-6, IL-8, VEGF) in the acute phase response and tissue healing has been shown to have a stimulatory effect on the growth of minimal residual disease. Altogether, despite that complete surgical resection offers the only chance for long-term survival in many solid organ cancers, surgery may come at a cost due to major changes in the perioperative period. Therefore, the perioperative period, which is at present almost unexploited, may also represent a window for novel therapeutic opportunities. Bearing this in mind, further characterization and quantification of circulating tumour cells (CTC) in the perioperative phase might help us find new therapeutic targets and predictive markers specific for the haematogenous metastatic route [5]. Despite the fact that specific isolation of CTC is a difficult pursuit, quantification of CTC has been found to possess significant prognostic value in numerous solid epithelial tumours [6-8]. Tumour cells have been detected and quantified in whole blood using the immunomagnetic EpCAM-based CellSearch^® ^system (Johnson & Johnson). The epithelial cell adhesion molecule (EpCAM) protein is commonly expressed on normal epithelial and overexpressed on malignant cells in a subset of human carcinomas [9]. For some authors the sensitivity of the CellSearch^® ^system, a slide-based cell counting technique has been a subject of concern. This likely reflects the fragility of CTC [10]. In contrast, molecular quantitative detection assays such as real-time qRT-PCR have much higher sensitivity. Real-time qRT-PCR has never been explored to detect EPCAM mRNA in patients with PDAC.

The primary aim of the current study was to prospectively study the value of a real-time RT-PCR assay for EpCAM detection in the peripheral blood and peritoneal cavity of patients undergoing pancreatectomy for PDAC.

Our secondary aim was to study correlations between CTC detection and clinical or pathological variables.

## Methods

### Patients

Between September 2004 and July 2006 we prospectively included 40 patients undergoing pancreatic resection (PR) for PDAC, 8 patients with unresectable PDAC, 10 patients with chronic pancreatitis and 3 healthy volunteers.. Pathological diagnosis was confirmed by microscopic evaluation of the resected specimen or tumour biopsy for unresectable cancers. Patients with pancreatic cancer were classified according to the 7^th ^edition of the AJCC TNM cancer staging system [11]. Of all resectable PDAC, 2 (5%) had stage Ib, 11 (27.5%) had stage IIa, 26 (65%) had stage IIb and one patient had stage IV disease.

Approval was obtained from the local ethical committee prior to the start of patient recruitment. Written informed consent was obtained from all included patients.

### Surgery

Two experienced hepatobiliary surgeons (RA and BT) performed all surgical procedures. A right para-aortic lymph node dissection was carried out routinely for staging purpose in pancreatic head tumours and followed by pancreaticoduodenectomy in case no metastatic lymph nodes were found at frozen section pathology. In the case of metastatic para-aortic lymph nodes a pancreaticoduodenectomy was not performed. The surgical resection margins of the common hepatic duct and the pancreatic transection surface were systematically examined on intra-operative frozen sections.

### Samples

EDTA-treated venous blood samples (10 ml) were obtained in patients undergoing PR before surgery (PB, n = 40, see Table 1), immediately after surgery at skin closure (POB, n = 40), at postoperative day 1 (D1B, n = 35), day 7 (D7B, n = 39) and after 6 weeks (6WB, n = 34). Blood samples were taken via a central venous line (except in healthy volunteers) in order to avoid contamination with skin epithelial cells. Peripheral blood mononuclear cells (PBMC) were isolated after erythrocyte lysis with 10 ml ammonium-chloride lysis buffer, resuspended, pooled and washed in Ca^2+^/Mg^2+ ^-free phosphate buffered saline. Next, cells were lysed and homogenized in RLT buffer (Qiagen) following the manufacturers instructions.

**Table 1:** Clinical and pathological characteristics of included patients.

	**EpCAM (+)** **N = 10**	**EpCAM (-)** **N = 30**	**Total** **N = 40**	**p-value**
	
**Age (±SD)**	56.3 (±10.0)	65.7 (±9.5)	63.3 (±10.3)	0.01
				
**Gender (M:F)**	5:5	18:12	23:17	NS
				
**pG**				NS
**1**	0	3 (10.0%)	3 (7.5%)	
**2**	2 (20%)	12 (40.0%)	14 (35.0%)	
**3**	8 (80%)	15 (50.0%)	23 (57.5%)	
				
**pT**				NS
**1**	0	2 (6.7%)	2 (5.0%)	
**2**	0	4 (13.3%)	4 (10.0%)	
**3**	10 (100%)	24 (80.0%)	34 (85.0%)	
				
**pN**				NS
**0**	3 (30.0%)	10 (33.0%)	13 (32.5%)	
**1**	7 (70.0%)	20 (67.0%)	27 (67.5%)	
				
**AJCC stage**				NS
**Stage IA**	0	2 (6.7%)	2 (5.0%)	
**Stage IB**	3 (30.0%)	8 (26.7%)	11 (27.5%)	
**Stage IIA**	0	1 (3.3%)	1 (2.5%)	
**Stage IIB**	7 (70.0%)	18 (60.0%)	25 (62.5%)	
**Stage III**	0	0	0	
**Stage IV**	0	1 (3.3%)	1 (2.5%)	
				
**ECLNI**				NS
**0**	7 (70.0%)	27 (90.0%)	34 (85%)	
**1**	3 (30.0%)	3 (10.0%)	6 (15%)	
				
**Total LN count**	16.5 (10 - 39)	17 (6 - 35)	17 (6 - 39)	NS
				
LVI *				NS
**0**	3 (33.3%)	20 (69.0%)	12 (31.6%)	
**1**	6 (66.7%)	9 (31.0%)	26 (68.4%)	
				
**PNI**				NS
**0**	1 (10.0%)	7 (23.3%)	8 (15%)	
**1**	9 (90.0%)	23 (76.7%)	32 (85%)	

NS: non-significant; pG: tumour differentiation grade; pN: lymph node status; ECLNI: Extracapsular lymph node involvement; LVI: lymphovascular invasion; PNI: perineural invasion.

* In 2 patients the presence of LVI was not evaluated.

Peritoneal lavage was performed in patients undergoing PR prior to exploration of the abdominal cavity (PP) and prior to laparotomy closure (POP). A volume of 500 ml of sterile isotonic sodium chloride solution was instilled and 40 ml was removed after irrigation. These samples were centrifuged at 1500 rpm for 10 minutes at 4°C in a swing-bucket centrifuge. The supernatant was removed and the sediment was diluted and washed with 10 ml of erythrocyte lysis buffer. Next, cells were counted, lysed and homogenized in RLT buffer (Qiagen). All cell lysates were stored at -80°C until total RNA was extracted.

Tumour (T) and surrounding non-tumoural control samples (P) were immediately stored in RNAlater^® ^(Qiagen) and transferred for storage to -80°C according to the manufacturers' instructions. Haematoxylin and eosin stains were made from each tumour sample to verify that the sample contained more than 70% tumour and from control pancreatic tissue to confirm its non-cancerous histology. In 16/40 patients RNA was extracted from the T and P samples.

### RNA extraction and cDNA synthesis

Total RNA from blood and peritoneal lavage fluid was extracted using the RNeasy kit (Qiagen). Total RNA was isolated from tissue sections using a protocol combining Trizol/chloroform extraction, followed by column chromatography with the RNeasy Mini kit (Qiagen). RNA integrity was quantified spectrophotometrically (Genequant). Copy DNA was synthesised from 4 μg of total RNA.

### Real-time RT-PCR assay

EpCAM and control gene Beta-glucuronidase (GUS) primers and probes were designed to span exon-intron boundaries. Amplification, detection and quantification were performed with the TaqMan™ ABI Prism 7700 sequence detection system (Applied Biosystems) and have been previously described [12]. Copy DNA was diluted to obtain a starting amount of 100 ng mRNA. Each sample was analyzed in duplicate. Copy numbers of the target template were quantified in peripheral blood and peritoneal fluid through generation of standard curves by serial dilution of plasmids for EpCAM and the housekeeping gene β-glucuronidase (GUS) (10^5 ^- 10^1 ^plasmid copies). All measured copies for EpCAM were normalized (i.e. normalized copy number (NCN)) to the quantities obtained for GUS.

### Cut-off strategy

Due to the background transcription in the blood and peritoneal lavage samples of the control group a cut-off was determined to differentiate between EpCAM positivity and negativity. The samples that exceeded the upper limit of the 95% confidence interval of NCN of EpCAM in the control group were defined as EpCAM mRNA positive.

### Follow-up

Follow-up data were recorded from the patient's medical records and completed by a telephone survey performed on the 1^st ^of April 2010, contacting the patient's general practitioner and/or oncologist. Postoperative mortality was defined as in-hospital mortality as from any postoperative complication. Cancer-specific survival (CSS) was defined as the time period from the date of surgery until any death after prior exclusion of postoperative deaths. Disease-free survival (DFS) was defined as the time interval between surgical resection and first recurrence of disease. First recurrence was classified as recurrence *in loco*, distant recurrence, or a combination of both. Median postoperative follow-up was 24.0 months (range: 0.7 - 41.3).

### Statistics

Statistical calculations were carried out using JMP version 8.0.1 for Mac (SAS).

The Student t test was performed to compare continuous data. The Fisher's exact test was used to compare categorical data whenever appropriate. The McNemar's test was used to compare paired proportions. The associations between EpCAM positivity was examined for the following clinical and histological parameters: age (years), gender (M/F), tumour differentiation (pG), lymph node metastasis (pN), tumour depth (pT), resection margin positivity (R), perineural invasion (PNI), lymphovascular invasion (LVI), extracapsular lymph node involvement (ECLNI) and total lymph node counts. Survival rates for CSS and DFS were estimated according to the Kaplan-Meier method using Log-Rank statistics for comparison. Two-sided P-values ≤ 0.05 were considered statistically significant.

## Results

### Primary tumour versus surrounding non-tumoural pancreatic tissue

Median (range) NCN (×1000) for EpCAM was 11800.1 (4357.7 - 39142.8) versus 7552.5 (475.2 - 13765.5) in tumour and non-tumoural surrounding pancreatic tissue respectively (p = 0.014). No significant correlations were found between pT, pG, pN, ECLNI, LVI, R and EpCAM expression in tumour or non-tumoural surrounding tissue. EpCAM expression was significantly increased in tumours with perineural invasion (p = 0.050).

### Perioperative detection of EpCAM in blood

**Figure 1:**
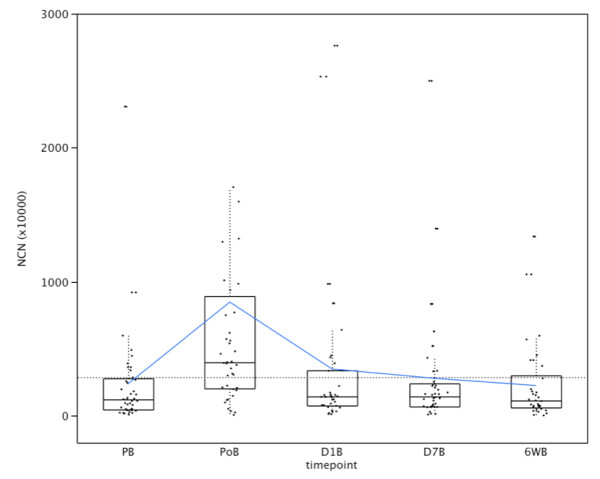
**Haematogenous cancer cell dissemination in patients undergoing pancreatic resection for PDAC**. Quantification of EpCAM transcripts in blood using real-time RT-PCR. The postoperative amounts are only significantly higher for PoB versus PB (p < 0.0001). PB: preoperative, PoB: postoperative; D1B: day after surgery; D7B: 7th day following surgery, 6WB: 6 weeks after surgery.

NCN were significantly higher in preoperative blood (PB) of unresectable PDAC versus resectable PDAC, with a median (range) of 149.3 (77.8 - 2855.6) NCN (×10^4^) versus 122.3 (9.5 - 2307.1) respectively (p = 0.014). EpCAM detection in blood increased significantly immediately after resection of PDAC with a median (range) of 397.7 (6.67 - 6891.9) NCN (×10^4^) (p = 0.001) (Figure 1). In contrast, no significant difference was found in NCN of PB between resectable PDAC and non-tumoural controls (benign pancreatic disease and HV) with a median (range) of 122.3 (9.5 - 2307.1) NCN (×10^4^) versus 157.1 (16.5 - 483.4) respectively (p = 0.60).

The upper limit of the 95% confidence interval of NCN (×10^4^) in preoperative blood of the control samples was 285.4. After dichotomization, preoperative blood was EpCAM(+) in 10/40 (25%) patients compared to 27/40 (67.5%) patients immediately after pancreatic resection (p < 0.0001).

For D1B, D7B and after 6 weeks, EpCAM positivity in blood was seen in 10/35 (28.6%), 9/39 (23.1%) and 8/34 (23.5%) patients undergoing PR respectively. In unresectable PDAC blood was EpCAM (+) in 2/8 (25%) patients.

**Figure 2:**
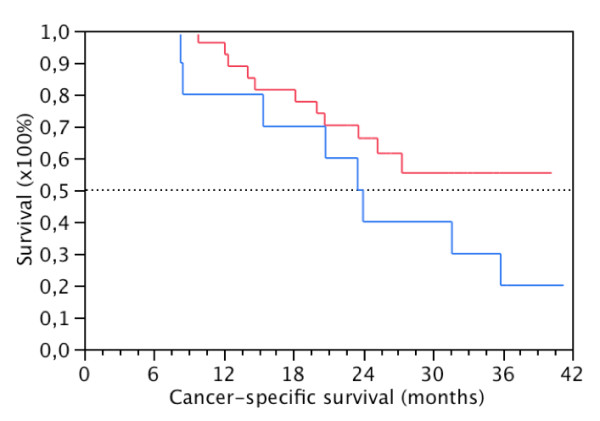
**Cancer-specifc survival rates by EpCAM-positivity**. EpCAM (+) (N = 10, blue) and EpCAM (-) (N = 28, red) patients in preoperative blood (PB) following pancreatic resection for PDAC after exclusion of postoperative mortality (p = 0.17, Log-Rank).

**Figure 3:**
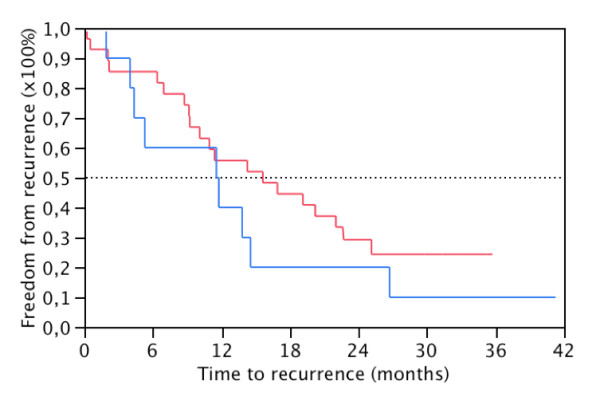
**Time-to-recurrence rates by EpCAM-positivity**. EpCAM (+) (N = 10, blue) and EpCAM (-) (N = 28, red) patients in preoperative blood (PB) following pancreatic resection for PDAC after exclusion of postoperative mortality (p = 0.28, Log-Rank).

No significant associations were found for preoperative (Figure 2 and 3) and postoperative (PoB, D1B, D7B and 6WB) EpCAM positivity in blood and CSS or DFS. Also, no significant associations were found for preoperative EpCAM positivity and pG, pT, pN, PNI, LVI, ECLNI and total lymph node counts. (Table 1) Finally, EpCAM expression in the primary tumour did not correlate with EpCAM positivity in preoperative blood (data not shown).

### Peritoneal detection of EpCAM

**Figure 4:**
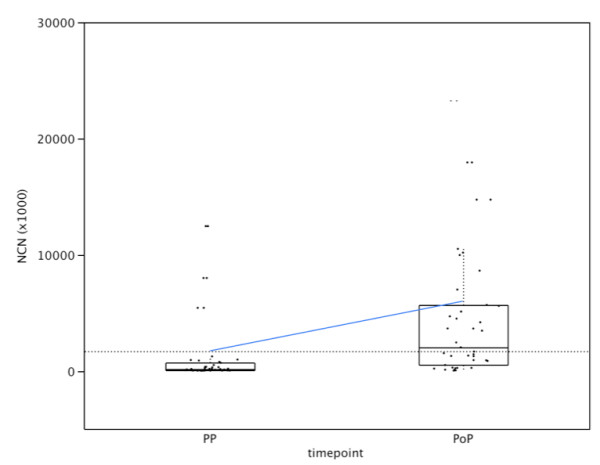
**Peritoneal cancer cell dissemination in patients undergoing pancreatic resection for PDAC**. Quantification of EpCAM transcripts in peritoneal lavage fluid using real-time RT-PCR. The postoperative amounts are significantly higher (p = 0.0001). *PP: preoperative, PoP: postoperative*.

No significant difference was observed in EpCAM NCN in preoperative peritoneal lavage fluid between resectable (N = 39) and unresectable (N = 8) PDAC despite a median (range) NCN (×10^3^) of 161.5 (3.39 - 31855.4) and 1539.6 (23.5 - 15059.3) respectively (p = 0.49). Similarly, no significant difference was found in EpCAM counts in preoperative peritoneal lavage specimens between resectable PDAC and non-tumoural controls (benign pancreatic disease). On the other hand, a significant difference in NCN was found between preoperative and postoperative peritoneal lavage specimens (p = 0.001) (Figure 4).

The upper limit of the 95% confidence interval of NCN (×10^3^) in preoperative peritoneal lavage specimens of the control samples was 1636.8. After dichotomization, preoperative lavage fluid was EpCAM(+) in 4/39 (10.3%) patients who underwent pancreatic resection compared to 21/39 (53.8%) patients immediately after pancreatic resection (p < 0.0001).

No significant associations were found for preoperative and postoperative (PoB, D1B, D7B and 6WB) EpCAM positivity in peritoneal lavage fluid and CSS or DFS (data not shown). Similar to EpCAM positivity in preoperative blood, no significant associations were found for preoperative EpCAM positivity in peritoneal lavage fluid and pG, pT, pN, PNI, LVI, R, ECLNI and total lymph node counts. Finally, EpCAM expression in the primary tumour did not correlate with EpCAM positivity in the preoperative peritoneal lavage fluid (data not shown).

## Discussion

The current study is the first using an EpCAM-based real-time RT-PCR assay to detect, quantify and evaluate the prognostic effect of circulating tumour cells in peripheral blood and peritoneal lavage fluid in any gastrointestinal cancer. Only one other research group studied the diagnostic potential of quantitative RT-PCR by detecting disseminated tumour cells based on cytokeratin-19 mRNA in blood, bone marrow and peritoneal lavage in patients with ductal adenocarcinoma of the pancreas [13].

Our study shows that peri-operative detection of EpCAM is not associated with worse oncological outcome. Nevertheless, significantly higher NCN were found in preoperative blood for unresectable versus resectable PDAC (p = 0.014). EpCAM detection was higher immediately after surgical resection (PoB), to decrease almost to preoperative levels as of postoperative day 1. Despite a significant increase in EpCAM counts and EpCAM positivity in the postoperative blood and peritoneal lavage fluid, no adverse effect on DFS or CSS could be demonstrated.

Several explanations can be proposed to elucidate these findings.

First, preoperative CTC could rather reflect intermittent shedding of tumour cells than true metastatic potential of the primary tumour. Moreover, it is well recognized that this shedding already commences at the early stages of tumour growth. Metastasis is an inefficient process where circulating malignant cells are eliminated almost immediately from the blood. A very large proportion - maybe the far majority - of exfoliated pancreatic tumour cells enter the peripheral circulation via the portal circulation and do not survive past the liver (i.e. first-pass effect). Accordingly, in our study the highest CTC load in peripheral blood was noted immediately after surgery, to quickly decrease by the first postoperative day. Thereafter the proportion of EpCAM-positive patients remained similar to the preoperative state. These findings are in line with previously published results that exfoliated tumour cells are almost completely cleared from the blood within 24 hours [14]. Moreover, the majority of cells that survive, arrest and extravasate in lymph nodes, lungs, liver and bone marrow will remain dormant for many years and may never cause relapse of cancer.

Second, it is well known that over 80% of patients suffering from PDAC present with unresectable disease due to the presence of metastases or local extension. However, to what extent the patients with potentially resectable PDAC have distant micrometastasis at presentation is unknown. As these distant sites also contribute to the pool of CTC, quantification of cancer cell dissemination might not be such a good surrogate of metastatic potential of the primary tumour but rather of total tumour load.

Third, growing evidence supports the importance of tumour cell heterogeneity [15]. Indeed, not all tumour cells have equal phenotypes. The capacity of a tumour to grow and propagate could be dependent on a subset of cells with the capacity of self-renewal and differentiation, often termed "cancer stem cells" or "cancer-initiating cells" [16]. According to this cancer stem cell theory only a proportion of cancer cells, i.e. cancer stem cells are able to proliferate extensively and form new tumours. Both the concept of tumour cell heterogeneity and the cancer stem cell hypothesis put a caveat on the perception of metastasis as a pure stochastic phenomenon. In the future, quantification with high specificity of the 'driving' cancer cell population in the peripheral blood or peritoneal cavity of pancreatic cancer patients, may prove to have far better prognostic value than quantifying all circulating cancer cells [6].

A fourth explanation is that all EpCAM-based detection systems could be associated with downregulation of epithelial markers - and consequently also EpCAM - in circulating tumour cells in the course of epithelial-mesenchymal transition (EMT) [17]. Despite a significant overexpression of EpCAM in primary PDAC compared to surrounding non-tumoural pancreatic tissue [9], EpCAM could still be downregulated in CTC. Nevertheless, the EpCAM-based semi-automated CellSearch^® ^(Johnson & Johnson) system has been found to bear significant prognostic potential. In patients with metastatic breast [7] and colorectal cancer [18], CTC levels were strong predictors for both progression-free survival and overall survival and complemented the results of conventional medical imaging. One must ascertain that in contrast with our single marker real-time RT-PCR assay, the CellSearch^® ^system also checks potential tumour cells for cytokeratin positivity, CD45 negativity and nuclear staining with DAPI and consequently should be considered a multimarker assay. Therefore, it is probably safer to state that any single-marker assay and a fortiori any non-cancer specific single-marker real-time RT-PCR study has limited biomarker potential.

A fifth explanation could be the high background expression of EpCAM mRNA in both chronic pancreatitis and healthy volunteers. Indeed, immunohistochemical studies have shown increased EpCAM in chronic pancreatitis. It is very likely that after and even before surgical resection for chronic pancreatitis, many different cell types including pancreatic epithelial cell types enter the blood circulation or peritoneal cavity and result in EpCAM detection. In this regard, circulating epithelial cells have been detected in the blood samples of patients undergoing colonic resection for benign conditions [19]. Yet, another explanation may be illegitimate transcription of EpCAM in haematopoietic cells, i.e. a low amount of tissue specific gene transcription is present in non-specific tissues as the result of minimal activation of promoters by ubiquitous transcription factors [20]. This phenomenon is more often encountered when RNA is isolated from the total white blood cell fraction (and not first exclude granulocytes) to detect CTC.

In our study EpCAM expression in primary PDAC samples was significantly higher in the presence of perineural invasion. Nevertheless, higher EpCAM expression was not associated with worse oncological outcome, despite that perineural invasion is a well-established prognostic factor in PDAC [21].

We are aware of a few shortcomings in the present study. The total sample size is relatively small with only 48 patients with PDAC included. This small sample size may result in both type I (false positive, e.g. the difference in EpCAM mRNA expression in unresectable versus resectable patients) and type II (false negative) errors. The detection of disseminated tumour cells with real-time RT-PCR depends on a number of steps including collection and treatment of the sample, cell separation protocol, chosen antibodies, and the number of analyzed cells. Ideally, the target gene is expressed only by tumour cells and not by surrounding cells in the examined compartment (i.e. the blood and peritoneal cavity in our study). However, a high background expression of EpCAM mRNA was also found in both chronic pancreatitis and healthy volunteers, necessitating application of a cut-off to discriminate between EpCAM-positivity and negativity. Similarly in colorectal cancer, the use of CK19, CK20 and other cytokeratins as a marker in the assessment of CTC in peripheral blood has been questioned primarily on the basis of high background expression in healthy volunteers [22]. Also, the ideal enrichment strategy (e.g. red blood cell lysis, density centrifugation, magnetic bead separation) and RNA extraction technique is still under debate, as undoubtedly both influence background expression. In several peritoneal lavage samples and some blood samples, the total starting amount of extracted RNA was less than 4 μg. After cDNA synthesis, the mixture was further diluted to obtain a concentration of 100 ng RNA per reaction for all samples. Nevertheless, different total starting amounts of RNA may result in varying sensitivity of the real-time RT-PCR assay for the detection of EpCAM in these samples. It is unclear to what extent our results have been influenced by this. However, since background expression in control samples was high, we believe that the assay is more likely to lack specificity than sensitivity.

## Conclusions

Circulating tumour cell load measured by real-time RT-PCR for EpCAM was increased significantly in the immediate postoperative period. However, perioperative cancer cell dissemination does not seem to be associated with worse prognosis in PDAC and should not be seen as a purely stochastic phenomenon. We believe that further study is necessary to clarify the complex interactions between disseminating tumour cells, the immune system, the acute phase response and healing process during the perioperative period.

## List of abbreviations

CTC: circulating tumour cells; PDAC: pancreatic ductal adenocarcinoma; RT-PCR: reverse transcriptase-polymerase chain reaction; EpCAM: Epithelial Cell Adhesion Molecule; EpCAM(+): EpCAM-positivity; EpCAM(-): EpCAM-negativity; PR: pancreatic resection; T: tumour samples; P: non-tumoural control samples; NCN: normalized copy numbers; PB: preoperative blood; PoB: postoperative blood, immediately after surgical resection; D1B: postoperative day 1; D7B: postoperative day 7, 6W at 6 weeks, CSS: cancer-specific survival; DFS: disease-free survival; pG: tumour differentiation; pN: lymph node metastasis; pT: tumour depth; R: resection margin positivity; PNI: perineural invasion; LVI: Lymphovascular invasion; ECLNI: extracapsular lymph node involvement

## Competing interests

The authors declare that they have no competing interests.

## Authors' contributions

GS participated in the study design, carried out the molecular assays, gathered the raw data, performed the statistical analysis and drafted the manuscript. TR carried out the pathological examination. JVP, FH and RA participated in the design of the study and helped with data gathering. BT conceived of the study, and participated in its design and coordination and helped to draft the manuscript. All authors read and approved the final manuscript.

## Pre-publication history

The pre-publication history for this paper can be accessed here:

http://www.biomedcentral.com/1471-2407/11/47/prepub
